# Four-junction superconducting circuit

**DOI:** 10.1038/srep28622

**Published:** 2016-06-30

**Authors:** Yueyin Qiu, Wei Xiong, Xiao-Ling He, Tie-Fu Li, J. Q. You

**Affiliations:** 1 Department of Physics, Fudan University, Shanghai 200433, China; 2 Beijing Computational Science Research Center, Beijing 100193, China; 3 School of Science, Zhejiang University of Science and Technology, Hangzhou, Zhejiang 310023, China; 4 Institute of Microelectronics, Department of Microelectronics and Nanoelectronics and Tsinghua National Laboratory of Information Science and Technology, Tsinghua University, Beijing 100084, China

## Abstract

We develop a theory for the quantum circuit consisting of a superconducting loop interrupted by four Josephson junctions and pierced by a magnetic flux (either static or time-dependent). In addition to the similarity with the typical three-junction flux qubit in the double-well regime, we demonstrate the difference of the four-junction circuit from its three-junction analogue, including its advantages over the latter. Moreover, the four-junction circuit in the single-well regime is also investigated. Our theory provides a tool to explore the physical properties of this four-junction superconducting circuit.

Superconducting quantum circuits based on Josephson junctions exhibit macroscopic quantum coherence and can be used as qubits for quantum information processing (see, e.g., refs 1, 2, 3, 4, 5, 6, 7, 8, 9). Behaving as artificial atoms, these circuits can also be utilized to demonstrate novel atomic-physics and quantum-optics phenomena, including those that are difficult to observe or even do not occur in natural atomic systems^10^. As a rough distinction, there are three types of superconducting qubits, i.e., charge^1^,^2^, flux^4^,^11^ and phase qubits^5^,^6^,^12^. In the charge qubit, where the charge degree of freedom dominates, two discrete Cooper-pair states are coupled via a Josephson coupling energy^1^,^2^. In contrast, the phase degree of freedom dominates in both flux^11^ and phase qubits^5^,^6^.

The typical flux qubit is composed of a superconducting loop interrupted by three Josephson junctions^11^. Similar to other types of superconducting qubits, it exhibits good quantum coherence and can be tuned externally. Recent experimental measurements^9^ showed that the decoherence time of the three-junction flux qubit can be longer than 40 *μ*s. Due to the convenience in sample fabrication (i.e., the double-layer structure fabrication by the shadow evaporation technique^13^), a superconducting loop interrupted by four Josephson junctions was also used as the flux qubit. The experiments^14^ showed that this four-junction flux qubit behaves similar to the three-junction flux qubit. Also, two four-junction flux qubits were interacting experimentally via a coupler^15^, similar to the interqubit coupling mediated by a high-excitation-energy quantum object^16^. The theory of the three-junction flux circuit with a static flux bias was well developed^17^, but a theory for the four-junction circuit lacks because adding one Josephson junction more to the superconducting loop makes the problem more complex.

In this paper, we develop a theory for the four-junction circuit with either a static or time-dependent flux bias. In addition to the similarity with the three-junction circuit, we demonstrate the difference from the three-junction circuit due to the different sizes of the two smaller Josephson junctions in the four-junction circuit. We find that the four-junction circuit with only one smaller junction has a broader parameter range to achieve a flux qubit in the double-well regime than the three-junction circuit. Moreover, for the four-junction circuit with two identical smaller junctions, the circuit can be used as a qubit better than the three-junction circuit, because it becomes more robust against the state leakage from the qubit subspace to the third level. This can be a useful advantage of the four-junction circuit over the three-junction circuit when used as a qubit. Also, we study the four-junction circuit in the single-well regime, which was not exploited before. Our theory can provide a useful tool to explore the physical properties of this four-junction superconducting circuit.

## Results

### The total Hamiltonian of the four-junction superconducting circuit

**Figure 1:**
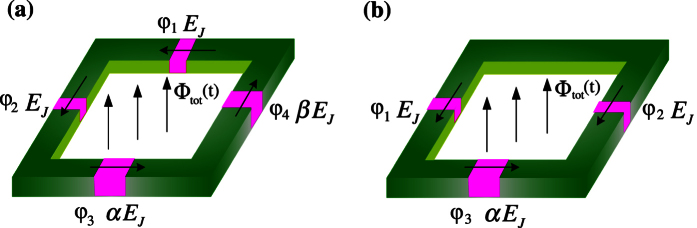
Schematic diagram of the considered superconducting circuits. (**a**) Superconducting loop interrupted by four Josephson junctions and pierced by a total magnetic flux, Φ_tot_(*t*), which includes the externally applied flux and the inductance-induced flux. Here two of the four junctions have identical Josephson coupling energy *E*_*J*_ and capacitance *C*. Among other two junctions, one has Josephson coupling energy *αE*_*J*_ and capacitance *αC*, and the other has Josephson coupling energy *βE*_*J*_ and capacitance *βC*, with 0 < *α*, *β* < 1. (**b**) Superconducting loop interrupted by three Josephson junctions and pierced by a total magnetic flux Φ_tot_(*t*), where two junctions have identical Josephson coupling energy *E*_*J*_ and capacitance *C*, while the third one has Josephson coupling energy *αE*_*J*_ and capacitance *αC*, with 0 < *α* < 1. In both (**a**,**b**), each red component denotes the thin insulator layer of a Josephson junction, and an arrow along the loop denotes the assigned direction of the phase drop across the corresponding Josephson junction. Note that each phase drop can be chosen along either the clockwise or counter-clockwise direction, but once the direction is fixed, the phase drop is positive along it.

Let us consider a superconducting loop interrupted by four Josephson junctions and pierced by a magnetic flux [see Fig. 1(a)], where the first and second junctions have identical Josephson coupling energy *E*_*J*_ and capacitance *C* (i.e., *E*_*Ji*_ = *E*_*J*_ and *C*_*i*_ = *C*, with *i* = 1, 2), while the third and fourth junctions are reduced as *E*_*J*3_ = *αE*_*J*_, *E*_*J*4_ = *βE*_*J*_, *C*_3_ = *αC*, and *C*_4_ = *βC*, with 0 < *α*, *β* < 1. The phase drops *φ*_*i*_ (*i* = 1, 2, 3, 4) through these four Josephson junctions are constrained by the fluxoid quantization


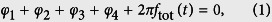


where 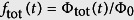, with Φ_tot_(*t*) being the total magnetic flux in the loop (which includes the externally applied flux, either static or time-dependent, and the inductance-induced flux owing to the persistent current in the loop) and Φ_0_ = *h*/2*e* being the flux quantum.

The kinetic energy of the four-junction circuit is the electrostatic energy^18^ stored in the junction capacitors, which can be written as


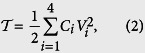


where 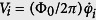 is the voltage across the *i*th junction. Using the the fluxoid quantization condition in Eq. (1), we can rewrite the kinetic energy as


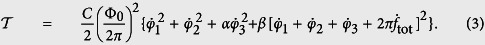


We introduce a phase transformation


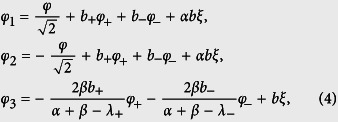


where


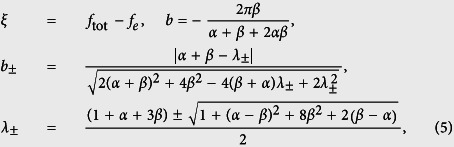


with 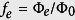 being the reduced static magnetic flux applied to the superconducting loop. The electrostatic energy 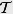 can then be converted to a quadratic form


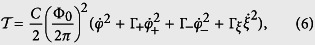


where


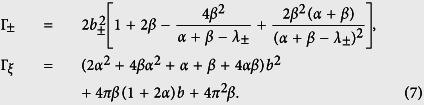


The total Josephson coupling energy of the four-junction circuit is


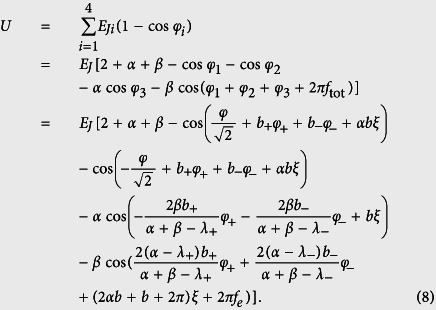


Also, there is the inductive energy due to the inductance *L* of the superconducting loop^19^:


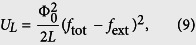


where the reduced externally-applied magnetic flux *f*_ext_ can generally be written as a sum of the static and time-dependent fluxes, i.e., *f*_ext_ = *f*_*e*_ + *f*_*a*_(*t*), with *f*_*a*_(*t*) ≡ Φ_*a*_(*t*)/Φ_0_ being the reduced time-dependent magnetic field applied to the four-junction loop. When including this inductive energy, the total potential energy of the four-junction circuit is written as


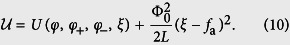


The Lagrangian of the four-junction circuit is


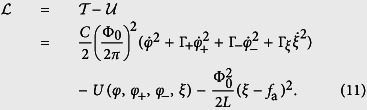


where we assign *φ*, *φ*_±_, and *ξ* as the canonical coordinates. The corresponding canonical momenta 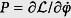, 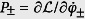, and 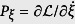 are


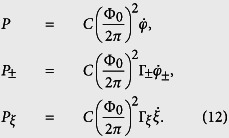


Therefore, the Hamiltonian of the four-junction circuit is given by


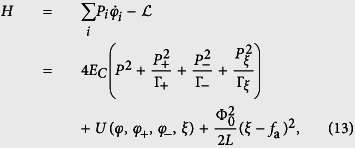


where *E*_*C*_ = *e*^2^/(2*C*) is the single-particle charging energy of the Josephson junction. In comparison with the previous work in ref. 17 for the three-junctions flux qubit, a new degree of freedom *ξ* is included in the Hamiltonian, so that the Hamiltonian can also apply to the case when the superconducting loop contains a time-dependent magnetic flux.

### The reduced Hamiltonian of the four-junction superconducting circuit

The total Hamiltonian of the four-junction circuit can be rewritten as


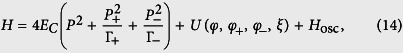


where


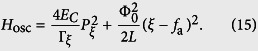


Quantum mechanically, the canonical momenta can be written as 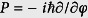, 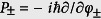, and 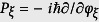 in the canonical-coordinate representation.

Note that the Hamiltonian *H*_osc_ in Eq. (15) can be rewritten as


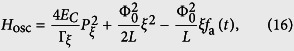


i.e., a harmonic oscillator driven by a time-dependent magnetic flux *f*_a_(*t*). The angular frequency of this harmonic oscillator is


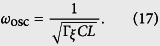


With the parameters achieved in experiments for the flux qubit^14^,^20^, *α* ~ 0.7, *C* ~ 8 fF, and *L* ~ 10 pH. Moreover, *β* ~ *α*, so *ω*_osc_/2*π* ~ 1 × 10^3^ GHz. For the four-junction flux qubit, the energy gap Δ between the lowest two levels is typically Δ ~ 1–10 GHz^14^,^15^, which is much smaller than *ω*_osc_/2*π* ~ 1 × 10^3^ GHz. Usually, the time-dependent magnetic flux *f*_a_(*t*) applied to the four-junction loop is a microwave wave with *ω*_*a*_/2*π* ~ 1–10 GHz, which is also much smaller than *ω*_osc_/2*π*. Because Δ ≪ *ω*_osc_/2*π* and the flux *f*_a_(*t*) is also very off resonance from the harmonic oscillator (i.e., *ω*_*a*_ ≪ *ω*_osc_), the oscillator is nearly kept in the ground state at a low temperature. Then, using the adiabatic approximation to eliminate the degree of freedom of the oscillator, the Hamiltonian of the four-junction circuit can be reduced to


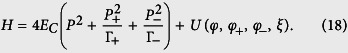


Also, both *L* and the persistent current *I* of the superconducting loop are small, so that^17^ *IL*/Φ_0_ ~ 10^−3^. This inductance-induced flux is much smaller than the externally applied magnetic flux *f*_ext_ = *f*_*e*_ + *f*_*a*_(*t*). Therefore, the total flux *f*_tot_ can also be approximately written as *f*_tot_ ≃ *f*_*e*_ + *f*_*a*_(*t*).

Below we first study the static-flux case, i.e., only a static magnetic flux is applied to the four-junction loop. In this case, *f*_tot_ ≃ *f*_*e*_, so *ξ* ≃ 0. The phase transformation in Eq. (4) becomes


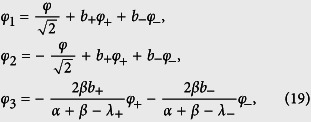


and the Hamiltonian of the four-junction circuit in Eq. (18) is further reduced to


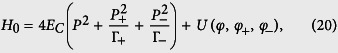


with 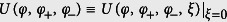.

**Figure 2:**
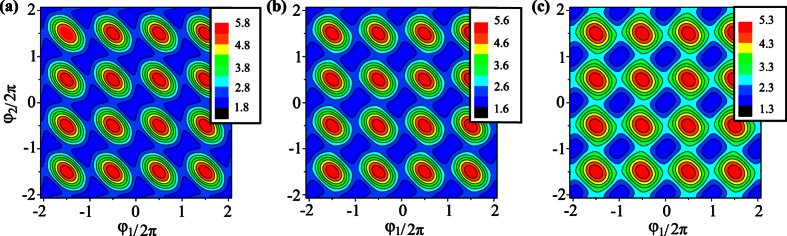
Contour plots of the potential *U*(*φ*_1_, *φ*_2_, *φ*_3_) at *φ*_3_ = 0 and *f*_*e*_ = 1/2. (**a**) *α* = 1, *β* = 0.8, (**b**) *α* = 1, *β* = 0.6, and (**c**) *α* = 1, *β* = 0.3.

Figure 2 shows the contour plots of the potential 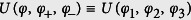 in the two-dimensional subspace spanned by *φ*_1_ and *φ*_2_ for *f*_*e*_ = 1/2, where *φ*_*i*_ (*i* = 1, 2, 3) are related to *φ* and *φ*_±_ by Eq. (19). For a three-junction flux qubit, *α* is usually in the range of 1/2 < *α* < 1. When 0 < *α* < 1/2, each double well in the potential is reduced to a single well^17^, so the flux qubit in the double-well regime is converted to a flux qubit in the single-well regime. For the four-junction circuit, there are wider ranges of parameters to achieve a flux qubit. For instance, in the case of three identical Josephson junctions (i.e., *α* = 1 and 0 < *β* < 1), when *β* > 1/3, the potential *U* (*φ*_1_, *φ*_2_, *φ*_3_) has two energy minima in the unit cell of three-dimensional periodic lattice at *φ*_1_ = *φ*_2_ = *φ*_3_ = ±φ* mod 2*π*, where


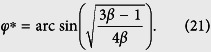


A flux qubit in the double-well potential can then be achieved in the parameter range of 1/3 < *β* < 1, which is broader than the range of 1/2 < *α* < 1 for the three-junction flux qubit. Figure 2(a,b) show a section of *U*(*φ*_1_, *φ*_2_, *φ*_3_) at *φ*_3_ = 0. Corresponding to the above-mentioned two minima, a figure-eight-shaped double well exists in each unit cell of the periodic lattice in the two-dimensional subspace. When *β* < 1/3, each figure-eight-shaped double well in the *φ*_3_ = 0 section of the potential is reduced to a single well [see Fig. 2(c)], with only one minimum in the unit cell at *φ*_1_ = *φ*_2_ = 0 mod 2*π*. This corresponds to a flux qubit in the single-well regime achieved in the four-junction superconducting circuit.

### Energy spectrum

The energy spectrum and eigenstates of the four-junction circuit are determined by


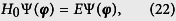


where ***φ*** ≡ (*φ*, *φ*_+_, *φ*_−_) = (*φ*_1_, *φ*_2_, *φ*_3_) is a three-dimensional vector in the phase space. Equation (22) is just like the quantum mechanical problem of a particle moving in a three-dimensional periodic potential *U*(***φ***). Thus, the solution of it has the Bloch-wave form


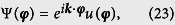


where ***k*** is a wavevector and *u*(***φ***) is a periodic function in the phases of *φ*_*i*_ (*i* = 1, 2, 3). Also, Ψ(***φ***) should be periodic in the phases of *φ*_*i*_. To ensure this, the wavefunction Ψ(***φ***) is constrained by ***k*** = 0. Then, Ψ(***φ***) can be written as


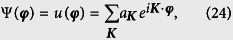


where ***K*** is a reciprocal lattice vector. Substituting Eq. (24) into Eq. (22), we then obtain an equation similar to the central equation in the theory of energy bands^21^. Numerically solving this equation, we can obtain the energy spectrum and eigenstates of the Hamiltonian *H*_0_.

For the three-junction flux qubit, an approximate tight-binding solution was obtained in ref. 17 by projecting the Schrödinger equation onto the qubit subspace, where the needed tunneling matrix elements were estimated using the WKB method. For the four-junction case, such an approximate tight-binding solution can also be derived, but it is difficult to calculate the tunneling matrix elements via the WKB method, because a three-dimensional potential is involved in the four-junction circuit. Thus, we resort to the numerical approach to solve the Schrödinger equation in Eq. (22). With this numerical approach, we can obtain the results for both the flux qubit and the three-level system.

**Figure 3:**
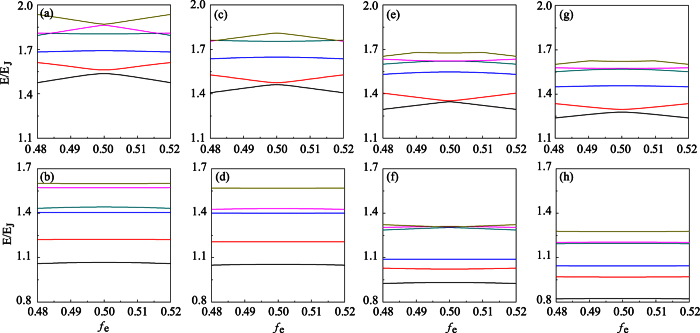
Energy spectra of the superconducting circuits versus the reduced static flux *f*_*e*_. (**a**) *α* = 0.7 and (**b**) 0.4 in the case of three-junction circuit; (**c**) *α* = 1 and *β* = 0.6, (**d**) *α* = 1 and *β* = 0.3, (**e**) *α* = *β* = 0.6, (**f**) *α* = *β* = 0.3, (**g**) *α* = 0.5 and *β* = 0.6, and (**h**) *α* = 0.2 and *β* = 0.3 in the case of four-junction circuit. In this figure and the following one, we choose *E*_*J*_ = 50*E*_*C*_.

Figure 3 shows the energy levels of the four-junction circuit versus the reduced static flux *f*_*e*_, in comparison with the three-junction circuit. In the case of four-junction circuit, when the lowest two or three levels are considered, the energy spectrum with *α* = 1 and *β* = 0.6 is similar to the energy spectrum with *α* = 0.7 in the case of three-junction circuit [comparing Fig. 3(c) with Fig. 3(a)]. Because the lowest two levels are well separated from other levels, both three- and four-junction circuits can be utilized as quantum two-level systems (i.e., flux qubits). In this case, the flux qubit can be modeled as


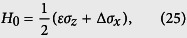


where the tunneling amplitude Δ corresponds to the energy difference between the two lowest-energy levels at *f*_*e*_ = 1/2, and *ε* = 2*I*_*p*_Φ_0_(*f*_*e*_ − 1/2) is the bias energy due to the external flux, with *I*_*p*_ being the maximal persistent current circulating in the loop. Here the maximal persistent current *I*_*p*_ can be approximately calculated as^17^ 
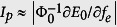
 at a value of *f*_*e*_ considerably away from *f*_*e*_ = 1/2, where *E*_0_ is the energy level of the ground state of the system. The Pauli operators *σ*_*z*_ and *σ*_*x*_ are represented using the two (i.e., the clockwise and counter-clockwise) persistent-current states. Moreover, similar to the three-junction circuit, the four-junction circuit can also be used as a quantum three-level system (qutrit) owing to the considerable separation of the third energy level from other higher levels as well. When reducing the smallest junction to, e.g., *β* = 0.3 in the four-junction circuit [see Fig. 3(d)], only the lowest two levels are well separated from other levels, similar to the case of three-junction circuit in Fig. 3(b) where *α* = 0.4. Now the double-well potential has been converted to a single well (see Fig. 2), so the circuit behaves as a flux qubit in the single-well regime. Compared to the flux qubits in Fig. 3(a,c), the energy levels in Fig. 3(b,d) are less sensitive to the external flux *f*_*e*_, so the obtained flux qubits in the single-well regime are more robust against the flux noise. However, because the smallest Josephson junction in the loop is further reduced, the charge noise may become important^22^. To suppress this charge noise, one can shunt a large capacitance to the smallest junction to improve the quantum coherence of the qubit^9^,^22^,^23^.

Furthermore, let us consider the four-junction circuit with two identical smaller Josephson junctions. In Fig. 3(e) where *α* = *β* = 0.6, the lowest two levels are also well separated from other levels, but the third level is not so separated from higher levels. Thus, from the energy-level point of view, this four-junction circuit can be better used as a flux qubit than a three-level system. In Fig. 3(f) where *α* = *β* = 0.3, the lowest three levels are well separated from other levels. It seems that the four-junction circuit can be better used as a three-level system. However, our calculations on transition matrix elements indicate that the circuit can still be better used as a qubit, because only the transition matrix element between the ground and first excited states is appreciably large (see the next section).

In addition, we further consider the case of two different smaller Josephson junctions (i.e., *α* ≠ *β*) in the four-junction circuit. In the double-well regime [see Fig. 3(g), where *α* = 0.5 and *β* = 0.6], the energy levels look similar to those in Fig. 3(e) and the lowest two levels can still be used as a qubit. Also, this qubit is less sensitive to the influence of the external magnetic field around the degeneracy point, because the energy levels are more flat than those in Fig. 3(e). In the single-well regime [see Fig. 3(h), where *α* = 0.2 and *β* = 0.3], the lowest three levels are well separated from the higher levels. Moreover, in addition to the transition matrix element between the ground and first excited states, the transition matrix element between the first and second excited states is also larger (see the section below). Therefore, in the single-well regime, the four-junction circuit in the case of *α* ≠ *β* can be better used as a quantum three-level system. This is different from the cases in Fig. 3(b,d,f).

### Transition matrix elements

Now we consider the time-dependent case with *f*_tot_(*t*) ≃ *f*_*e*_ + *f*_*a*_(*t*), i.e., in addition to a static flux *f*_*e*_, a time-dependent flux 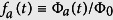 is also applied to the four-junction loop. In this case, *ξ* ≃ *f*_*a*_(*t*) when ignoring the very small inductance-induced flux. For a small enough time-dependent flux, only the first-order perturbation due to *ξ* needs to be considered in Eq. (18). Then, the Hamiltonian of the four-junction circuit in Eq. (18) can be expressed as


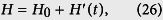


with *H*_0_ given in Eq. (20) and


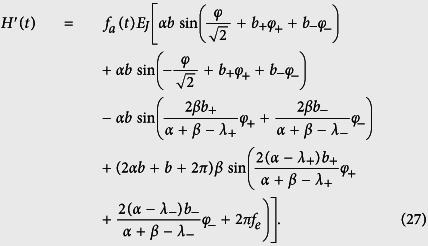


The time-dependent perturbation 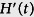 can be rewritten as


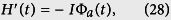


where


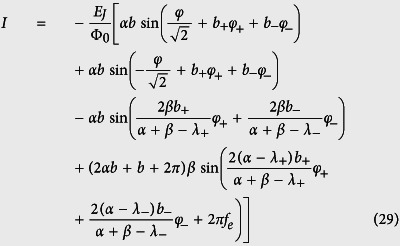


is the current in the superconducting loop. Because


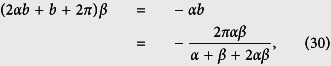


we can express the current *I* as


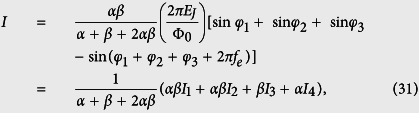


where *I*_*i*_ = *I*_*c*_ sin*φ*_i_, with *i* = 1, 2 and *I*_*c*_ = 2*πE*_*J*_/Φ_0_, *I*_3_ = *αI*_*c*_ sin*φ*_3_, and 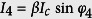 are Josephson supercurrents through the four junctions. The phase drops *φ*_*i*_ (*i* = 1, 2, 3) are related to *φ* and *φ*_±_ by Eq. (19), and *φ*_4_ is constraint by the fluxoid quantization condition in the static-flux case, i.e., 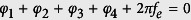.

Here we consider a microwave field with frequency *ω*_*a*_ applied to the superconducting loop. The time-dependent magnetic flux in the loop can be written as 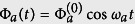. Then, with the current *I* available, the magnetic-dipole transition matrix elements are calculated by


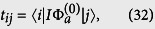


where |*i*〉 and |*j*〉 are eigenstates of the Hamiltonian *H*_0_ in Eq. (20).

**Figure 4:**
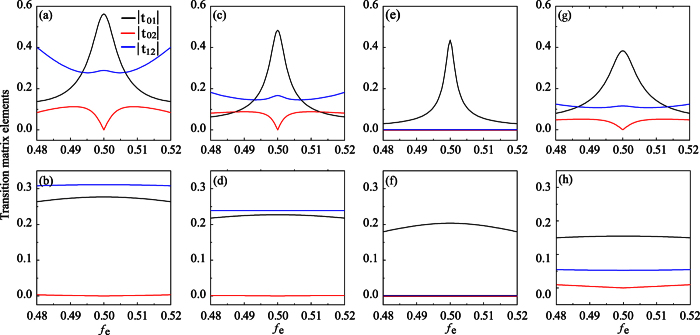
Transition matrix elements |*t*_01_|, |*t*_02_| and |*t*_12_| of the superconducting circuits (in units of 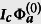) versus the reduced static flux *f*_*e*_. (**a**) *α* = 0.7 and (**b**) 0.4 in the case of three-junction circuit; (**c**) *α* = 1 and *β* = 0.6, (**d**) *α* = 1 and *β* = 0.3, (**e**) *α* = *β* = 0.6, (**f**) *α* = *β* = 0.3, (**g**) *α* = 0.5 and *β* = 0.6, and (**h**) *α* = 0.2 and *β* = 0.3 in the case of four-junction circuit.

Figure 4 shows the transition matrix elements |*t*_01_|, |*t*_02_|, and |*t*_12_| of the three- and four-junction circuits as a function of the reduced static flux *f*_*e*_, where the subscripts 0, 1 and 2 correspond to the ground state |0〉, the first excited state |1〉, and the second excited state |2〉 of the system, respectively. Similar to the three-junction circuit in Fig. 4(a) where *α* = 0.7, the four-junction circuit with *α* = 1 and *β* = 0.6 (i.e., there is only one smaller Josephson junction in the circuit) behaves as a ladder-type (namely, Ξ-type^24^) three-level system at *f*_*e*_ = 1/2, and a cyclic-type (Δ-type^25^) three-level system at *f*_*e*_ ≠ 1/2 [see Fig. 4(c)]. For the Ξ-type three-level system achieved when *f*_*e*_ = 1/2, the transition between the ground state |0〉 and the second excited state |2〉 is not allowed, which is analogous to a natural atom. However, for the Δ-type three-level system at *f*_*e*_ ≠ 1/2, all transitions among |0〉, |1〉 and |2〉 are allowed. This is different from a natural atomic system^25^. When the smallest Josephson junction is further reduced, |*t*_02_| is greatly suppressed. Now both three- and four-junction circuits behave more like a Ξ-type three-level system in the whole region of *f*_*e*_ shown in Fig. 4(b,d).

As for the four-junction circuit with two identical smaller Josephson junctions (*α* = *β*), while |*t*_01_| remains appreciably large, the transition between |0〉 and |2〉 as well as the transition between |1〉 and |2〉 are greatly reduced (i.e., |*t*_02_| ≈ 0 and |*t*_12_| ≈ 0) in the whole region of *f*_*e*_ shown in Fig. 4(e,f). Now, in either double- or single-well regime, the four-junction circuit can be well used as a qubit, because the state leakage from the qubit subspace to the third level is suppressed. This is an apparent advantage of the four-junction circuit over the three-junction circuit when used as a qubit.

When the two smaller Josephson junctions in the four-junction circuit become different (i.e., *α* ≠ *β*), in addition to |*t*_01_|, both |*t*_02_| and |*t*_12_| become nonzero except for the degeneracy point [see Fig. 4(g,h)]. This circuit behaves very different from the circuit with two identical smaller junctions [comparing Fig. 4(g) with Fig. 4(e), and comparing Fig. 4(h) with Fig. 4(f)], but it is similar to the three-junction circuit and the four-junction circuit with only one smaller junction [comparing Fig. 4(g) with Fig. 4(a,c), and comparing Fig. 4(h) with Fig. 4(b,d)]. However, when the distribution of the energy levels is also taken into account (see Fig. 3), the four-junction circuit with *α* ≠ *β* can be better used as a quantum three-level system (qutrit) in the single-well regime. This is very different from the three-junction circuit and the four-junction circuit with only one smaller junction, which can be better used as a qubit in the single-well regime. Therefore, as compared to the three-junction circuit, the four-junction circuit can provide more choices to achieve different quantum systems.

## Summary

We have developed a theory for the four-junction superconducting loop pierced by an externally applied magnetic flux. When the loop inductance is considered, the derived Hamiltonian of this four-junction circuit can be written as the sum of two parts, one of which is the Hamiltonian of a harmonic oscillator with a very large frequency. This makes it feasible to employ the adiabatic approximation to eliminate the degree of freedom of the harmonic oscillator in the total Hamiltonian. Also, this theory can be used to study the case when the applied magnetic-flux bias becomes time-dependent. In the case of static flux bias, the total Hamiltonian of the four-junction circuit is reduced to the Hamiltonian of the superconducting qubit. When the flux bias is time-dependent, the total Hamiltonian of the four-junction circuit can be reduced to the Hamiltonian of the superconducting qubit plus a perturbation related to the applied time-dependent flux. Then, we can calculate the energy spectrum and the transition matrix elements of the four-junction superconducting circuit.

In conclusion, we have studied the four-junction superconducting circuit in both double- and single-well regimes. In addition to the similarity with the three-junction circuit, we show the difference of the four-junction circuit from its three-junction analogue. Also, we demonstrate its advantages over the three-junction circuit. Owing to the one additional Josephson junction in the circuit, the physical properties of the four-junction circuit become richer than those of the three-junction circuit. For instance, in the case of four-junction circuit with only one smaller Josephson junction, the circuit has a broader parameter range to achieve a flux qubit in the double-well regime than the three-junction circuit does. Moreover, in the case of four-junction circuit with two identical smaller junctions, the circuit can be used as a qubit better than the three-junction circuit in both double- and single-well regimes. This is because among the lowest three eigenstates of the four-junction circuit, only the transition matrix element between the ground and first excited states is appreciably large, while other two elements become zero. These properties of the four-junction circuit can suppress the state leakage from the qubit subspace to the second excited state, and the circuit with these parameters is thus expected to have better quantum coherence when used as a qubit.

## Methods

### Three-junction circuit with a time-dependent magnetic flux

To compare with our four-junction results, we also consider a three-junction superconducting loop pierced by a time-dependent total magnetic flux Φ_tot_(*t*) [see Fig. 1(b)], because no explicit derivation exists in the literature for this time-dependent case. The directions of the phase drops *φ*_*i*_ (*i* = 1, 2, 3) through the three Josephson junctions are chosen as in ref. 17, which are constrained by the following fluxoid quantization condition:


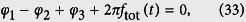


where 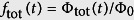. Here we assume that two larger junctions have identical capacitance *C* and coupling energy *E*_*J*_, while the smaller junction has capacitance *αC* and coupling energy *αE*_*J*_, with 0 < *α* < 1.

Similar to the four-junction circuit, we introduce a phase transformation


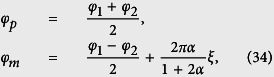


where 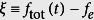, with 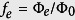 being the reduced static magnetic flux applied to the superconducting loop. The Hamiltonian of the three-junction circuit can be derived as


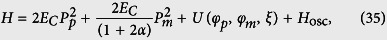


where *E*_*C*_ = *e*^2^/(2*C*),


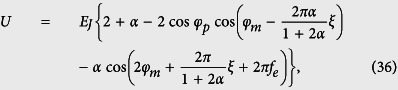


and


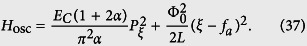


Quantum mechanically, the canonical momenta can be written as 
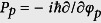
, 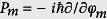, and 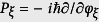 in the canonical-coordinate representation.

The angular frequency of the harmonic oscillator given in Eq. (37) is


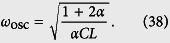


Using the parameters achieved in experiments^14^,^20^, we have *α* ~ 0.7, *C* ~ 8 fF, and *L* ~ 10pH, so one has *ω*_osc_/2*π* ~ 10^3^ GHz, which is much larger than the energy gap Δ ~ 1–10 GHz of the three-junction flux qubit (see, e.g., ref. 4). If the time-dependent magnetic flux is the usually applied microwave field, the oscillator can indeed be regarded as being in the ground state at a low temperature, as analyzed for the four-junction flux qubit in the main text. Then, the Hamiltonian of the three-junction circuit can be reduced to


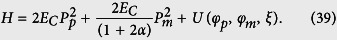


Because *L* is small in a three-junction flux qubit^17^, we can ignore the flux generated by the loop inductance. Thus, when only a static flux is applied to the loop, *f*_tot_(*t*) ≃ *f*_*e*_, i.e., *ξ* ≃ 0. The phase transformation in Eq. (34) becomes


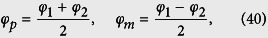


and the Hamiltonian of the circuit in Eq. (39) is reduced to


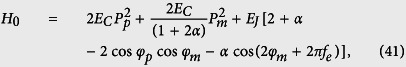


which is the Hamiltonian of the three-junction flux qubit derived in ref. 17.

For the time-dependent case with 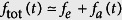, 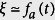, where 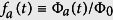 is the reduced time-dependent magnetic flux applied to the three-junction loop. When the time-dependent magnetic flux is small enough, only the first-order perturbation due to *ξ* needs to be considered, and the Hamiltonian of the circuit in Eq. (39) can be expressed as


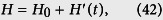


with *H*_0_ given in Eq. (41) and 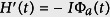, where


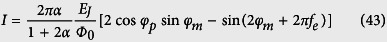


is the current in the three-junction loop^26^. Using Eq. (40) and the fluxoid quantization condition in the static-flux case (i.e., 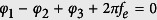), the current *I* can also be rewritten as


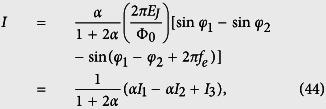


where *I*_*i*_ is the Josephson supercurrent through each junction. Moreover, as in Eq. (32), the magnetic-dipole transition matrix elements are calculated by 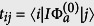, where |*i*〉 and |*j*〉 are eigenstates of the Hamiltonian *H*_0_ in Eq. (41).

## Additional Information

**How to cite this article**: Qiu, Y. *et al.* Four-junction superconducting circuit. *Sci. Rep.* **6**, 28622; doi: 10.1038/srep28622 (2016).
